# Prolonged Catatonia and Severe Malnutrition in Adolescents Following Bullying-Triggered Stress: A Dual Case Report

**DOI:** 10.7759/cureus.83415

**Published:** 2025-05-03

**Authors:** Emmanuel Annor, Venkata S Lokireddy, Dhanalakshmi Ramasamy

**Affiliations:** 1 Child and Adolescent Psychiatry, Lehigh Valley Health Network, Allentown, USA

**Keywords:** adolescents, bullying, catatonia, malnutrition, stress

## Abstract

Catatonia is a complex neuropsychiatric syndrome characterized by motor, behavioral, and affective disturbances. Though traditionally associated with schizophrenia, it is now recognized across a range of psychiatric and medical conditions. Catatonia is often underrecognized in the pediatric population, and bullying-induced psychological trauma is a largely overlooked trigger.

This case report presents two adolescents who developed prolonged catatonia and severe malnutrition following intense bullying-triggered stress.

The first case involves a 17-year-old female with developmental delay who developed severe catatonia and malnutrition following a school bullying incident. Despite initial treatment with benzodiazepines and antidepressants, her condition necessitated electroconvulsive therapy (ECT) after recurrent episodes and prolonged functional decline. The second case involves a 13-year-old female with DiGeorge syndrome who developed catatonia following emotional and physical bullying. Her symptoms partially responded to benzodiazepines, antipsychotics, and supportive care.

Both patients exhibited classic catatonic features, including mutism, rigidity, and refusal to eat, and required multidisciplinary interventions. Pharmacological management with lorazepam was central, with ECT proving critical in the first case due to poor response to medication. Nutritional rehabilitation and trauma-informed psychosocial support were also essential to recovery.

These cases emphasize the role of bullying-related trauma as a significant yet underrecognized trigger for pediatric catatonia. Early recognition and intervention, including using ECT, are vital to recovery. Increasing awareness, implementing preventive strategies, and conducting future research on school bullying, its effects, and its connection to catatonia may help decrease the occurrence of severe psychiatric outcomes.

## Introduction

Catatonia is a complex neuropsychiatric syndrome characterized by motor, behavioral, and affective abnormalities [1]. Prompt recognition and accurate diagnosis are essential, as catatonia can be life-threatening if misidentified as other medical emergencies, such as serotonin syndrome or neuroleptic malignant syndrome, both of which present with altered consciousness and autonomic dysfunction but have distinct underlying mechanisms and treatment approaches [2]. The latest edition of the Diagnostic and Statistical Manual of Mental Disorders (DSM-5-TR) acknowledges catatonia as a syndrome that may manifest in conjunction with primary psychiatric disorders, neurological diseases, and various other medical conditions. Although catatonia is not categorized as an independent disorder, it is classified as a specifier - 'Catatonia associated with another mental disorder' - to denote its co-occurrence with mood disorders, schizophrenia, and medical conditions [3].

While the exact pathophysiology of catatonia is not yet fully understood, research indicates a dysfunction in the right medial and lateral orbitofrontal cortex. Moreover, disturbances in the neurotransmission of gamma-aminobutyric acid (GABA), glutamate, serotonin, and dopamine have been linked to catatonic symptoms, affecting both motor and behavioral expressions [4]. The prevalence of pediatric catatonia in inpatient settings ranges from 0.6% to 17%. Pediatric catatonia predominantly occurs during puberty. Unlike adults, it is more prevalent in males, with a male-to-female ratio of about 2:1 [5]. To conduct a thorough evaluation of a young person presenting with catatonic symptoms, a multidisciplinary approach is crucial. It is necessary to perform both a detailed psychiatric evaluation and a neurological assessment. A vital assessment aspect involves a complete medical workup addressing differential diagnoses. This should encompass blood tests, including basic hematologic and metabolic assessments, a toxicology screen, brain imaging, autoimmune antibody tests, and any other investigations suggested by the clinical examination [5,6].

The Bush-Francis Catatonia Rating Scale (BFCRS) includes 23 items that evaluate the presence and severity of various symptoms. These symptoms, such as rigidity, negativism, mutism, and refusal to eat, can lead to significant malnutrition risks and prolonged hospital stays, which may cause nutritional deficiencies. The Pediatric Catatonia Rating Scale (PCRS) is a modified version of the Bush-Francis Catatonia Rating Scale (BFCRS), validated for research in children and adolescents in inpatient settings. While its validation studies confirmed its psychometric properties, such as internal consistency and construct validity, they primarily focused on its reliability as a measurement tool rather than assessing its effectiveness as a clinical screening instrument for this population [7]. Treatment typically includes benzodiazepines like lorazepam, which are effective in reducing catatonia symptoms. If benzodiazepines are ineffective, electroconvulsive therapy (ECT) is a safe alternative [8,9]. ECT, with reversible side effects like memory loss and confusion, also promotes neuroprotection, increasing hippocampal and amygdala gray matter volume, potentially reversing catatonia symptoms. ECT shows 76% and 92% response rates in youth with catatonia. However, challenges such as legal restrictions, inadequate clinical trials, community resistance, and stigma limit its use in children and adolescents [9,10].

Traumatic factors like deprivation, abuse, and trauma can trigger catatonia in pediatric patients, as Dhossche et al. discuss in their exploration of trauma in institutionalized children, including physical trauma and attachment issues, highlighting the interconnectedness of these experiences and their impact on children's mental health [10]. Early deprivation is linked to biological changes, including endocrine, immune, electrophysiological, and neuropsychological alterations. Central GABA function, crucial for HPA stress response regulation, significantly contributes to catatonia [11].

Bullying is a common form of peer violence defined as aggressive, intentional acts that are carried out repeatedly by an individual or group against a victim who is unable to defend themselves easily. It includes verbal attacks, physical behaviors, social aggression, and increasingly, cyberbullying. Bullying affects 20-25% of youth as perpetrators or victims, with 4-9% frequently involved. It peaks at ages 12-15 and declines in high school, shifting from physical to relational forms, with boys more often physically victimized. Reviewed school-based anti-bullying programs demonstrate a 20-23% reduction in bullying incidents and a 17-20% drop in victimization. Bullying in schools is associated with negative health effects such as increased absenteeism, diminished academic performance, elevated anxiety and depression, suicidal thoughts, and psychotic symptoms when compared to peers who are not victimized [12-14]. Nevertheless, research connecting bullying to catatonia remains limited.

This report details two adolescents who developed catatonia after separate incidents of bullying, thereby adding to the literature on stress-induced pediatric catatonia caused by bullying.

## Case presentation

Case 1

A 17-year-old girl presented to our emergency department (ED) on June 2, 2024, with reduced oral intake, limited verbal communication, and an unintended weight loss of 18 pounds, along with declining developmental milestones. She displayed low energy, a depressed mood, anhedonia, lack of motivation, increased sleepiness, and severe withdrawal from social and family interactions. Furthermore, she experienced visual hallucinations of “bugs” in the environment and auditory hallucinations of voices suggesting danger to her family and saying she was “bad.” Although she did have occasional periods of greater alertness and interaction, according to her mother, these moments had become less frequent over several weeks.

Symptoms began following a bullying incident at school in March 2024, when she reported to her mother that two girls had followed her to the restroom, cursing and banging on the stall door. Her mother had noticed a significant decline in the patient’s mental health and functioning after the patient’s birthday in April 2024. This deterioration became increasingly evident as the weeks passed, leading the patient to need help with activities of daily living (ADLs) such as brushing her teeth, showering, and combing her hair. The mother reported the patient had expressed feelings of distress with statements such as “School bad,” “bad teachers,” and “naked phones,” raising the mother’s concerns about the patient’s overall well-being. The mother described the patient at baseline as verbal and interactive, with some developmental delays, and noted that she had a healthy appetite. The patient has a past medical history of mild intellectual disability and scoliosis and a surgical background of reconstructive otoplasty. She had no documented psychiatric history, no history of psychoactive substance use, no family history of mental illness, and no recent medical illnesses or fevers.

The physical examination in the ED indicated that the patient was not in acute distress and had clear tympanic membranes. She had a regular heart rhythm, no murmurs, clear lungs, and a soft, non-tender abdomen. Neurologically, pupils and eye movements were normal. Muscle tone was normal, with no tremors or clonus, and reflexes were 2+ in the upper extremities and 1+ in the lower extremities. The patient had a depressed and anxious mood, a flat affect, psychomotor retardation, and minimally answered questions. She exhibited negativism, waxy flexibility, and episodes of staring. A clinical diagnosis of catatonia was established, and the BFCRS was utilized to evaluate severity and monitor progression. Her initial score was 18 (Table 1).

**Table 1:** Bush-Francis Catatonia Rating Scale scores over the trajectory of care Note: Scores > 2 on at least two of the first 14 screening items indicate a positive catatonia diagnosis. The Bush-Francis Catatonia Rating Scale (BFCRS) assesses severity by scoring symptoms from 0 to 3 across 23 items. Based on cumulative scores, catatonia is categorized as mild (1-9), moderate (10-19), or severe (≥20). The patient showed improvement after acute treatment with lorazepam challenge; however, she had catatonia symptoms after tapering off lorazepam and showed gradual improvement post-ECT. ECT: electroconvulsive therapy

Date administered	Phase of Treatment	Score
June 3, 2024	Initial admission (Pre-lorazepam)	18
June 21, 2024	Post-lorazepam	15
June 22, 2024	Pre-ECT	22
September 24, 2024	Post-ECT	10

It was initially uncertain whether the etiology was medical or psychiatric. Possible diagnoses included encephalitis, neuropsychiatric lupus, Wilson’s disease, vitamin deficiencies, nutritional encephalopathy, mood- or psychotic-related disorders, and seizures.

**Figure 1:**
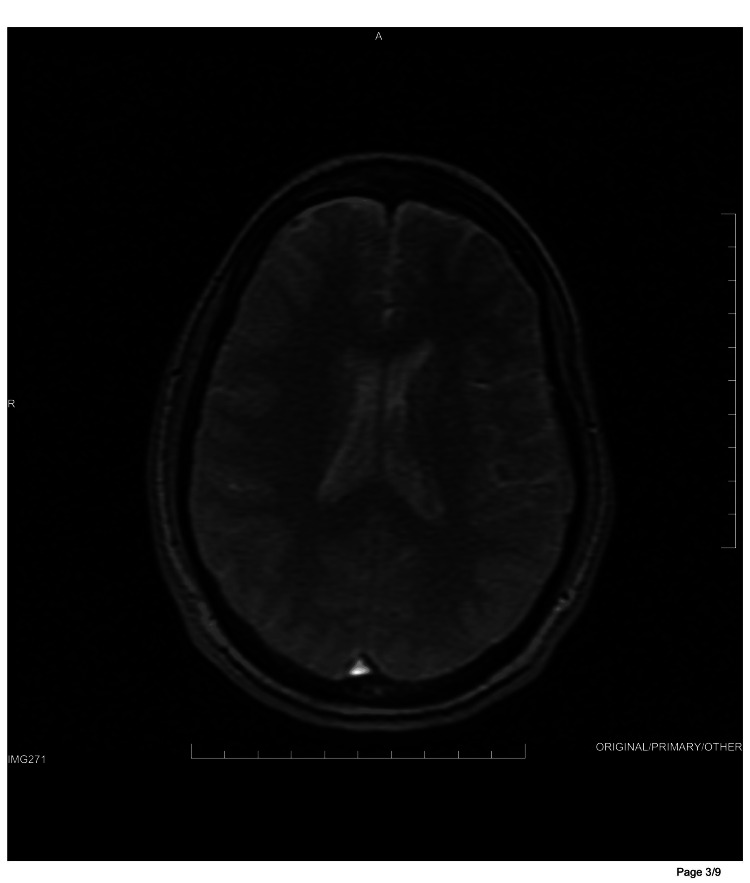
MRI showed no acute infarction or hemorrhage No diffuse brain atrophy in the prefrontal cortex and basal ganglia, no diffuse white matter lesions affecting the frontal and temporal lobes, and no alterations in the caudate nucleus and putamen

A thorough medical evaluation was conducted to identify any underlying medical conditions. This assessment included a brain MRI, both with and without contrast (Figure 1), which showed no acute infarction or hemorrhage, and an EEG was normal. Additional lab tests indicated low levels of magnesium and 25-hydroxyvitamin D, while phosphorus and vitamins B1 and B12 were within normal limits (Table 2).

**Table 2:** Initial laboratory workup on presentation at the ED The initial workup revealed low levels of magnesium, ceruloplasmin, and vitamin D (25-OH), while vitamin B1, vitamin B12, antinuclear antibodies, 24-hour urinary copper excretion, and ammonia remained within the normal range.

PARAMETERS	PATIENT VALUES	REFERENCE VALUE
Complete Blood Count		
Hemoglobin	14.9	11.9 -14.8 g/dl
White Blood Cell Count	7.6	3.8 - 10.4 thou/cmm
Platelets	343	158 - 362 thou/cmm
Mean Corpuscular Volume	89	83 - 98 fl
Mean Corpuscular Hemoglobin	30.9	27.6 - 33.3 pg
Mean Corpuscular Hemoglobin Concentration	34.9	32.5 - 35.2 g/dl
Red Cell Distribution Width	14	11.4 - 13.5 %
Complete Metabolic Panel		
Sodium	140	135 - 145 mmol/l
Potasiium	3.7	3.5 - 5.2 mmol/l
Calcium	10.4	8.5 - 10.1 mg/dl
Chloride	102	100 - 109 mmol/l
Creatinine	0.91	0.50 - 0.80 mg/dl
Blood Urea Nitrogen	15	7.0 - 20 mg/dl
Carbon Dioxide	23	21 - 31 mmol/l
Glucose	71	65 - 99 mg/dl
Liver Function Test		
Albumin	5.2	3.8 - 5.2 g/dl
Protein, total	8.3	6.2 - 7.7 g/dl
Total Bilirubin	0.8	0.2 - 0.8 mg/dl
Aspartate Aminotransferase	13	12.0 - 24.0 U/l
Alanine Aminotransferase	10	< 56 U/l
Alkaline Phosphatase	55	52.0 - 239 U/l
Differentials		
Ammonia	40	18 - 72 umol/l
Ceruloplasmin	18	22 - 61 mg/dl
24 Hour Urinary Copper Excretion	< 1	3.0 - 45.0 ug/d
Antinuclear Antibodies	Absent	<1.0 IU
Phosphorus	3.5	3.0 - 4.8 mg/dl
Magnesium	1.9	2.0 - 2.7 mg/dl
Vitamin B1	70	70 - 180 nmol/l
Vitamin B12	529	180 - 914 pg/ml
Vitamin D, 25-OH	15	30 - 100 ng/ml

The patient was admitted and initially prescribed 25 mg quetiapine orally at bedtime for psychosis symptoms, but it was discontinued after one dose due to sedation and urinary incontinence. The treatment was followed by risperidone 0.25 mg orally twice daily, which was also discontinued after two days due to suspicion of worsening catatonia. A diagnosis of catatonia linked to severe major depressive disorder (MDD) with psychosis due to stress or traumatic incidents was established. She was then started on lorazepam 1 mg/kg orally twice daily, which was titrated to three times daily. Fluoxetine 10 mg was prescribed to manage depression and anxiety symptoms. A nasogastric (NG) tube was placed on the fourth day of admission due to acute moderate protein-calorie malnutrition and poor oral intake.

After five days of treatment with lorazepam, the patient showed improvements in her mood and catatonic symptoms, tolerated oral intake, and gained 3 pounds. She was discharged on June 12, 2024, with fluoxetine at 10 mg daily and a tapering dose of lorazepam from 2.5 mg/day to 0.5 mg/day orally in divided doses for five days.

On June 21, 2024, toward the end of the outpatient lorazepam taper, the patient returned to the ED with worsening symptoms consistent with her previous presentation. She was nonverbal, exhibited psychosis with paranoia, and reported seeing “devil faces” and hiding under a table in her room. An NG tube was reinserted for feeding, the lorazepam dose was increased to 1 mg twice daily, and fluoxetine was continued at 10 mg daily. Her lorazepam dosage was gradually increased to 1.5 mg four times daily (total 6 mg daily maximum) until she was discharged on the same dose. Discussions regarding alternative treatment options (including ECT) were initiated with the family in case of inadequate improvement, and the patient was discharged on June 27, 2024, on fluoxetine 10 mg daily and lorazepam 1 mg four times daily with the recommendation of an outpatient psychiatrist titrating the medications.

In August 2024, the patient was readmitted with severe protein-calorie malnutrition and risk of refeeding syndrome due to ongoing catatonia and multiple instances of NG tube removal and medication non-compliance since her last hospitalization. The multidisciplinary care team included a social worker, pediatric surgeon, occupational therapist, pediatric physical therapist, speech therapist, and psychologist. Upon admission, lorazepam was restarted at 1.5 mg four times daily for two days, then adjusted to 2 mg every six hours (total 8 mg daily maximum), and an increased fluoxetine dose of 20 mg. Additionally, she received melatonin at a dose of 3-6 mg for insomnia. A gastrostomy tube (G-tube) was placed on September 5, 2024, to address her malnutrition. Olanzapine was initiated at 1.25 mg at bedtime via the G-tube to manage psychosis; however, it was discontinued after one dose due to excessive sedation. The patient exhibited minimal improvement despite increasing the doses of lorazepam. The patient’s mother agreed to proceed with ECT. Given the patient’s age and reduced capacity to assent due to her mental health condition, the team sought court approval for the ECT, which was granted on September 10, 2024, for 30 sessions at a frequency of three per week. Before the ECT sessions, the nighttime doses of lorazepam and melatonin were held.

**Figure 2:**
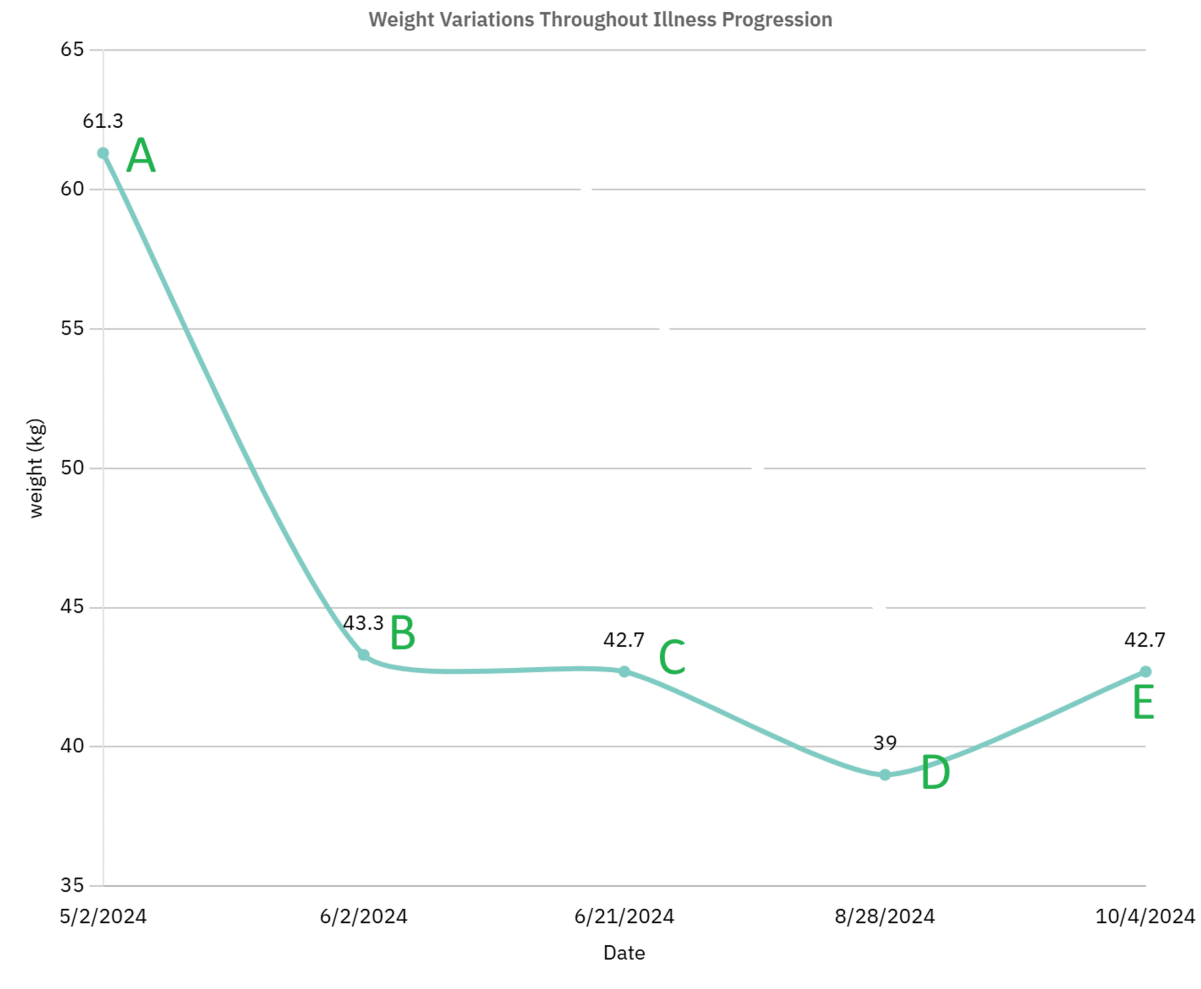
Changes in body weight over the course of illness: X-axis (date) and Y-axis (weight in kg) A – In May, the patient weighed 61.7 kg shortly after the mother noticed signs of decline. B – Upon presentation to the Emergency Department, the patient's weight had dropped sharply to 43.3 kg. Some improvement was observed following the lorazepam challenge and acute management. C – The patient initially maintained stability, but after lorazepam tapering, weight declined to 42.7 kg. D – Catatonic symptoms became refractory to lorazepam, leading to further weight loss to 39.0 kg, prompting the initiation of ECT. E – Post-ECT, the patient demonstrated gradual improvement, with weight returning to 42.7 kg. ECT: electroconvulsive therapy

The patient began showing improvements in mood and catatonic symptoms after the fifth ECT session, initially communicating through writing, maintaining conversations, interacting with family and staff, increasing oral intake, and gaining independence in ADLs. The fluoxetine dose was increased to 40 mg, aripiprazole was initiated at 2 mg daily and then adjusted to 4 mg to control psychosis, and lorazepam was gradually reduced from a maximum of 8 mg daily. Upon discharge on October 4, 2024, the patient had completed nine ECT sessions, and her medication doses were as follows: fluoxetine 40 mg, lorazepam 0.25 mg each morning, 0.50 mg at bedtime, and aripiprazole 4 mg. She also was set up for follow-up outpatient mental health care. Weight changes throughout her admission are illustrated in Figure 2.

Case 2

**Figure 3:**
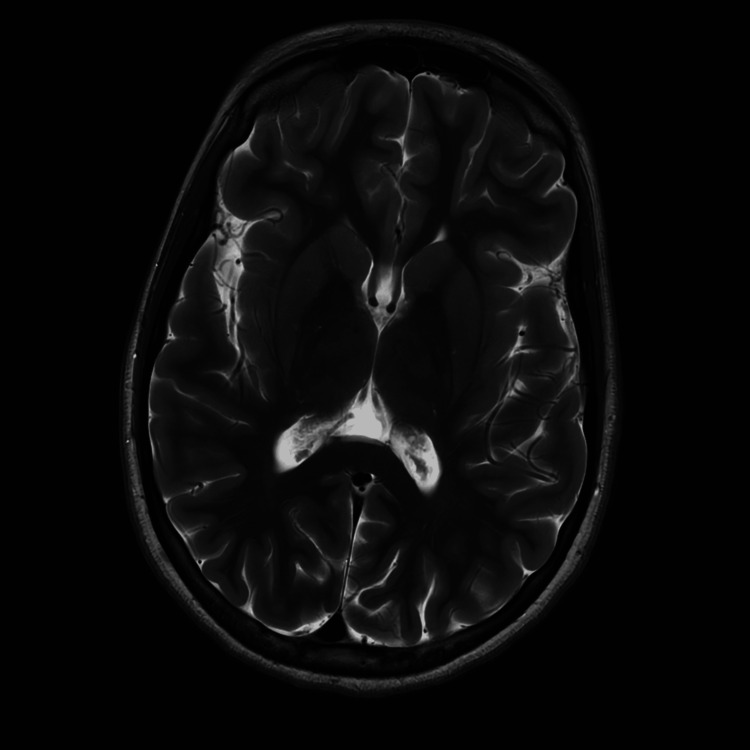
MRI of the brain with contrast showing no acute intracranial abnormality or pathologic enhancement

A 13-year-old girl with a medical history of DiGeorge syndrome, depression, and anxiety presented to the ED on September 8, 2024, exhibiting symptoms of catatonia, including mutism, rigidity, social withdrawal, paranoia, and a refusal to eat or drink. Symptoms began after the patient experienced physical and emotional bullying at the start of the school year in September 2024. The patient reported an injury to her right hand that required surgery. Following this, she became paranoid, refused to leave her home, and expressed fears that “some people were going to expose me online.” Since then, she reported having suicidal ideations without intent or plan. She reported experiencing visual and auditory hallucinations following the traumatic incidents. The patient had a history of bullying at school, no prior suicide attempts, no history of substance use, and no previous psychiatric hospitalizations. However, she had a previous ED visit after witnessing her older sister engaging in self-harming behavior and experiencing worsening anxiety and was referred to the Partial Hospitalization Program (PHP). The patient was discharged from the PHP in August 2024 on quetiapine 25 mg orally at bedtime. At baseline, the patient was described as an easygoing child who did not like to get into arguments. MRI of the brain, both with and without contrast, showed no acute intracranial abnormalities or pathological enhancement (Figure 3). Routine lab tests showed slightly elevated calcium and creatinine levels while remaining largely unremarkable (Table 3).

**Table 3:** Initial laboratory workup The initial workup revealed mildly elevated calcium and creatinine levels, while ethanol, beta-human chorionic gonadotropin serum, thyroid-stimulating hormone, acetaminophen, and salicylate remained within the normal range.

PARAMETERS	PATIENT VALUES	REFERENCE VALUE
Complete Blood Count		
Hemoglobin	13	11.9 -14.8 g/dl
White Blood Cell Count	7.7	3.8 - 10.4 thou/cmm
Platelets	234	158 - 362 thou/cmm
Mean Corpuscular Volume	88	83 - 98 fl
Mean Corpuscular Hemoglobin	29.9	27.6 - 33.3 pg
Mean Corpuscular Hemoglobin Concentration	34.2	32.5 - 35.2 g/dl
Red Cell Distribution Width	13.3	11.4 - 13.5 %
Complete Metabolic Panel		
Sodium	136	135 - 145 mmo/l
Potasiium	4.1	3.5 - 5.2 mmol/l
Calcium	10.3	8.5 - 10.1 mg/dl
Chloride	101	100 - 109 mmol/l
Creatinine	0.91	0.50 - 0.80 mg/dl
Blood Urea Nitrogen	22	7.0 - 20 mg/dl
Carbon Dioxide	22	21 - 31 mmol/l
Glucose	70	65 - 99 mg/dl
Liver Function Test		
Albumin	4.8	3.8 - 5.2 g/dl
Protein, total	9.1	6.2 - 7.7 g/dl
Total Bilirubin	1	0.2 - 0.8 mg/dl
Aspartate Aminotransferase	23	12.0 - 24.0 U/l
Alanine Aminotransferase	13	< 56 U/l
Alkaline Phosphatase	127	52.0 - 239 U/l
Differentials		
Ethanol	<10	< 10 mg/dl
Beta-HCG serum	<1	< 5 mIU/ml
Thyroid Stimulating Hormone	1.26	0.68 - 3.35 uIU/ml
Acetaminophen	<2	10.0 - 30.0 ug/ml
Salicylate	<2	15.0 - 30 mg/dl

Treatment began with lorazepam, initially at a dose of 0.5 mg, and then increasing to 1 mg twice daily, resulting in partial improvement and not requiring ECT treatment. Subsequently, the dosage of lorazepam was raised to 1 mg three times daily and then to 1.5 mg three times daily. At the same time, quetiapine was increased to 50 mg at bedtime to manage the underlying paranoia and mood symptoms. Quetiapine was later discontinued due to hypotension. Then, due to excessive sedation, the daily dose of lorazepam was reduced from a total of 4.5 mg to 2 mg daily, which necessitated further titration of the lorazepam dose.

To address her depression and anxiety, the patient was prescribed escitalopram 5 mg to be taken orally. Additionally, she received aripiprazole 2 mg orally to manage her underlying paranoia. Nutritional deficiencies were managed through NG feeding, which gradually transitioned to oral intake.

The patient improved after pharmacological and psychosocial interventions, including increased appetite, better verbal communication, and reduced paranoia. The patient was discharged on October 7, 2024, with prescriptions for aripiprazole 7.5 mg, escitalopram 5 mg, and lorazepam 1 mg to be taken orally four times daily. She was instructed to follow up at the PHP for ongoing trauma-focused outpatient therapy and medication management.

## Discussion

Table 4 compares the clinical and treatment characteristics of the two cases and highlights the link between severe malnutrition, psychosocial stress, and catatonia, emphasizing the importance of a multidisciplinary approach to diagnosis and treatment [6]. The patients exhibited classic symptoms of catatonia such as mutism, posturing, waxy flexibility, withdrawal, unresponsiveness, and significant nutritional depletion. A shared factor was psychological stress from bullying, which can provoke mood disorders and impair physical functioning, potentially leading to catatonia, particularly in those with pre-existing conditions. Research shows that trauma, particularly in individuals with PTSD, is associated with the onset of catatonia. Biles et al. suggest that catatonia may serve as a pathological response to trauma, with severe psychological distress potentially triggering its symptoms [15]. They found that trauma-induced decompensation can hinder functioning, resulting in severe malnutrition. Similarly, Nejati and Etches discovered that children with neurodevelopmental disorders are especially susceptible to catatonia after traumatic experiences [8]. These findings indicate that trauma can heighten both the risk and intensity of catatonia in vulnerable children. Collectively, these studies reinforce the link between psychological trauma and neuropsychiatric disorders like catatonia, aligning with the theory of an evolutionary-based freezing response to intense emotions [8,15].

**Table 4:** Comparison of clinical and treatment characteristics in bullying-triggered catatonia cases SSRI: selective serotonin reuptake inhibitor; SGA: second-generation antipsychotic; ECT: electroconvulsive therapy

Patient Factors	Case 1: 17-Year-Old Girl	Case 2: 13-Year-Old Girl
Underlying Condition	Developmental Delay	DiGeorge Syndrome
Trigger	Bullying	Bullying and past trauma
Symptoms	Catatonia, Malnutrition, Depression, Paranoia, Hallucinations	Catatonia, Refusal to eat, Depression, Paranoia, Hallucinations
Treatment	Benzodiazepines, ECT, Antidepressant (SSRI), Antipsychotics (SGA), Nutritional support, Therapy	Benzodiazepines, Antidepressant (SSRI), Antipsychotic (SGA), Nutritional support, Therapy
Outcome	Gradual improvement with ECT	Improvement without ECT

The patients showed positive responses to lorazepam treatment, confirming the diagnosis. A gradual reduction in BFCRS scores indicated clinical improvement (Table 1) following the administration of benzodiazepines. Due to the refractory nature of Case 1 to increasing doses of lorazepam, the next option for treating catatonia, ECT, was initiated. The patient demonstrated gradual improvements in weight (Figure 2) and verbal interaction. This supports the literature on reported response rates between 76% and 92% [9,10,16].

In both cases, a background of severe malnutrition increased the risk of developing catatonia. Inadequate nutrient intake, which leads to deficiencies in essential vitamins, magnesium, iron, and selenium, can result in cognitive impairments and weakness. These nutrients play vital roles in synthesizing and regulating neurotransmitters, providing antioxidants, modulating the immune system, and affecting serotonin levels and synaptic plasticity [17]. A 24-hour urinary copper excretion of <1 mg, combined with low ceruloplasmin levels, suggests a copper deficiency likely due to severe malnutrition in Case 1. EEG and neuroimaging were normal, revealing no underlying structural abnormality or seizures.

A crucial part of this case was differentiating nutritional encephalopathy from catatonia (Table 5). Encephalopathy usually showcases cognitive decline with systemic symptoms, while catatonia is mainly a neuropsychiatric syndrome defined by motor and behavioral disruptions. The lack of considerable metabolic disturbances, paired with a positive response to lorazepam, reinforced the diagnosis of catatonia instead of an encephalopathic condition. However, it is essential to consider how malnutrition may intensify catatonia. Table 5 illustrates the distinctions among encephalopathy, nutritional encephalopathy, and catatonia.

**Table 5:** Comparison of nutritional encephalopathy, general encephalopathy, and catatonia CT: computed tomography; MRI: magnetic resonance imaging; EEG: electroencephalogram

Feature	Nutritional Encephalopathy	General Encephalopathy	Catatonia
Cause	Deficiencies of essential nutrients (thiamine, vitamin B12, niacin, magnesium)	Various systemic conditions (hepatic, uremic, toxic, metabolic, infectious, anoxic)	Neurotransmitter dysregulation (GABA, dopamine, glutamate) triggered by psychiatric or medical conditions.
Clinical Symptoms	Confusion, memory loss, ataxia, neuropathy, ophthalmologic signs	Altered mental status, seizures, agitation, movement disorders, coma	Mutism, posturing, rigidity, waxy flexibility, echolalia, withdrawal
MRI findings	Hyperintensities in the thalamus, mammillary bodies, periaqueductal gray (Wernicke)	Diffuse cortical or subcortical changes, cerebral edema, metabolic abnormalities	Often normal, but subtle, frontal and basal changes may be seen
Diagnostic Approach	Vitamin and electrolyte panel, clinical response to supplementation	Blood tests (metabolic panel, ammonia, toxins), imaging (CT scan/MRI), EEG	Bush-Francis Catatonia Rating Scale (BFCRS), lorazepam challenge
Treatment	Nutritional supplementation (thiamine, B12, niacin), electrolyte correction	Treat underlying cause (dialysis for uremic encephalopathy, liver support for liver encephalopathy)	Benzodiazepines (lorazepam), electroconvulsive therapy (ECT) for severe cases

This case report provides insights into catatonia's relationship with severe malnutrition but acknowledges several limitations. The findings are limited by the small sample of two adolescent patients, affecting their generalizability. While the cases showed similar symptoms and treatment responses, a larger population is necessary for broader trend validation. The report primarily focuses on acute symptoms and initial treatment, lacking long-term follow-up on relapse rates and sustained cognitive function. Future studies should include longitudinal assessments to evaluate symptom persistence, novel drug delivery technologies that increase targeted delivery, bioavailability, and treatment outcomes in neuropsychiatric disorders, and more systematic research on the connection to trauma-induced catatonia. Psychosocial stress, particularly bullying, can complicate symptom onset interpretation. Although severe malnutrition contributed, it's unclear if the catatonic state stemmed from stress or metabolic dysfunction. Differentiating between psychiatric catatonia and metabolic encephalopathy emphasizes a multidisciplinary evaluation approach. The findings may not apply to other groups, such as adults or those with existing psychiatric disorders, as factors like diet, socioeconomic status, and medical access affect disease progression and treatment. Broader studies, including diverse age groups and demographics, are essential for understanding malnutrition's impact on catatonia. Despite limitations, this report highlights the importance of early recognition and intervention in catatonia, particularly for patients with nutritional deficits. Future research should explore the complex relationship between malnutrition and neuropsychiatric disorders to enhance diagnostic clarity and treatment outcomes.

## Conclusions

This case report illustrates the relationship between catatonia, severe malnutrition, and psychosocial stress, emphasizing the necessity for a multidisciplinary strategy for diagnosis and treatment. The cases show the urgent need for heightened awareness of bullying as a potential precipitant of catatonia in youth. Timely identification of catatonia and early intervention, including ECT when indicated, can be life-saving. Nutritional rehabilitation helped stabilize metabolic needs. Trauma-induced catatonia among adolescents underscores the need for evidence-based prevention programs aimed at reducing bullying and its psychological effects in school settings. Implementing effective anti-bullying strategies is crucial, as it may help prevent severe neuropsychiatric outcomes.

Clinicians must remain vigilant in recognizing signs of catatonia in school-aged children and adolescents experiencing bullying, withdrawal symptoms, and malnutrition, integrating psychiatric and medical care to maximize treatment outcomes.

## References

[1] Dhossche DM, Wachtel LE (2010). Catatonia is hidden in plain sight among different pediatric disorders: a review article. Pediatr Neurol.

[2] Elrad DR, Takahashi N, Walsh M (2025). Trauma-induced catatonia in pediatric patients: case series and literature review. Journ Child Adol Trauma.

[3] Tandon R, Heckers S, Bustillo J (2013). Catatonia in DSM-5. Schizophr Res.

[4] Burrow JP, Spurling BC, Marwaha R (2025). Catatonia. https://www.ncbi.nlm.nih.gov/books/NBK430842/.

[5] Cohen D, Nicolas JD, Flament MF (2005). Clinical relevance of chronic catatonic schizophrenia in children and adolescents: evidence from a prospective naturalistic study. Schizophr Res.

[6] Lahutte B, Cornic F, Bonnot O (2008). Multidisciplinary approach of organic catatonia in children and adolescents may improve treatment decision making. Prog Neuropsychopharmacol Biol Psychiatry.

[7] Benarous X, Consoli A, Raffin M, Bodeau N, Giannitelli M, Cohen D, Olliac B (2016). Validation of the Pediatric Catatonia Rating Scale (PCRS). Schizophr Res.

[8] Nejati N, Etches S (2024). Identifying and treating catatonia in children with neurodevelopmental disorders: a case series. J Can Acad Child Adolesc Psychiatry.

[9] Ridgeway L, Okoye A, McClelland I, Dhossche D, Kutay D, Loureiro M (2021). Case report: a case of pediatric catatonia: role of the lorazepam challenge test. Front Psychiatry.

[10] Pal R, Cheng T, Eddington S, Subramanian S, Wenzinger M, Cristancho P (2024). Use of electroconvulsive therapy in children and adolescents with catatonia--a case series. J ECT.

[11] Dhossche DM, Ross CA, Stoppelbein L (2012). The role of deprivation, abuse, and trauma in pediatric catatonia without a clear medical cause. Acta Psychiatr Scand.

[12] Herman JP, Cullinan WE (1997). Neurocircuitry of stress: central control of the hypothalamo-pituitary-adrenocortical axis. Trends.

[13] Menesini E, Salmivalli C (2017). Bullying in schools: the state of knowledge and effective interventions. Psychol Health Med.

[14] Olweus D (1978). Aggression in the schools: bullies and whipping boys. Hemisphere.

[15] Biles TR, Anem G, Youssef NA (2021). Should catatonia be conceptualized as a pathological response to trauma?. J Nerv Ment Dis.

[16] Ahmed GK, Elbeh K, Karim AA, Khedr EM (2021). Case report: catatonia associated with post-traumatic stress disorder. Front Psychiatry.

[17] Catatonia Disorder. (2024 (n.d). CatatoniaDisorder. Nutritional deficiencies associated with catatonic symptoms. https://catatoniadisorder.com/nutritional-deficiencies-and-catatonia/.

